# Implementation of malaria vector surveillance and insecticide resistance monitoring interventions in Nigeria

**DOI:** 10.1186/s41256-024-00397-4

**Published:** 2024-12-31

**Authors:** Abiodun Obembe, Adedayo O. Oduola, Adedapo Adeogun, Uwem Inyang, Tolulope Oyeniyi, Abiodun Olakiigbe, Ayodele Babalola, Petrus Inyama, Samdi Lazarus, Ifeanyi Okeke, Mary Esema, Okefu Oyale Okoko, Mamudu Omo-Eboh, Perpetua Uhomoibhi, Mohammed Bala, Samson Awolola

**Affiliations:** 1 Department of Zoology, Kwara State University, Malete, Nigeria; 2 Abt Associates- U. S. President’s Malaria Initiative Evolve Project Nigeria, Abuja, Nigeria; 3 Nigerian Institute of Medical Research, Lagos, Nigeria; 4 United States Agency for International Development/PMI, Abuja, Nigeria; 5 National Malaria Elimination Programme, Federal Ministry of Health, Abuja, Nigeria

## Abstract

Malaria vector surveillance is required to determine disease transmission dynamics, vector insecticide susceptibility status, suitable control strategies and impact of control interventions. However, capacity and resources for vector surveillance and insecticide resistance monitoring is often inadequate in most countries at risk of vector-borne diseases. Collaborations and linkages between malaria control policy makers and existing research institutions generating vector surveillance research data are often weak, thereby hindering the availability of data for decision-making. A national vector surveillance programme, mobilizing all stakeholders towards quality data generation and policy making, is required for effective data-driven country-wide vector control. This paper provides an analysis and suggested future directions for such synergized national malaria vector surveillance and insecticide resistance monitoring system currently being implemented by all research and policy stakeholders in Nigeria. The harmonized national vector surveillance system described here can be used as a model for the development or improvement of such structures in other countries with high malaria transmission risks.

## Background

The steady progress recorded in global malaria burden decline since 2000 ceased between 2015 and 2017 [1]. Current estimates of global disease incidence and mortality rates reveal threats of a malaria burden rebound [1–4], requiring strengthened intervention systems. The World Health Organization (WHO) African Region with an estimated 233 million malaria cases, accounted for 94% of the 249 million cases globally in 2022 [3]. In the WHO African Region, Nigeria alone has accounted for at least 25% of the global malaria cases since 2015 [4]. Bulk of the malaria burden decline from 2000 to 2015 has been attributed to intensified vector control using insecticide-treated nets (ITNs) and indoor residual spraying (IRS) with recommended insecticides [5].

Nigeria relies mainly on mass ITN distribution and replacement campaigns as an integral tool for malaria vector control in the country. Earlier, there were only pockets of un-coordinated vector control research activities mostly from south-western part of the country [6] with little or no link towards generating data required for programmatic malaria control decisions. Thus, the vector control intervention in the country were not evidence-based and the baseline entomological data for control performance assessment were not available. The unavailability of malaria entomological data to guide implementation of vector control strategies is often attributed to weak capacities in local control programmes [7]. National and subnational control programmes often have limited medical entomology personnel, access to training tools and infrastructure to support the fight against vector-borne disease. This restricts the ability to perform basic functions beyond vector control implementation, such as surveillance, monitoring and evaluation [8]. One previous effort to assess and build malaria vector research and control capacities in Nigeria, through the training of 23 participants from Nigerian higher institutions, identified the need for increased personnel access to malaria vector surveillance, research and control tools across the six geopolitical zones [7].

Sustained synergized collection of malaria vector surveillance (MVS) and insecticide resistance monitoring (IRM) information in each localized region of a country will require a well-funded system of recruitment, training and supervision of vector control personnel commissioned to generate current data for evidence-based national vector control decisions. Such system must include a collective data harvesting, harmonization, and evaluation mechanism geared towards arriving at the appropriate cost-effective vector control decisions for the country. Here, we examine the implementation of in-country coordinated system for malaria vector surveillance and insecticide resistance monitoring in Nigeria with suggestions on future directions.

## Frame work for the implementation of coordinated MVS and IRM programme in Nigeria

Nigeria is made up of 36 states and one federal capital territory (FCT). Considering the vast geographical size and epi-ecological diversity of the country, localized vector surveillance through the establishment of state-based sentinel sites was adopted for central harmonization and evidence-based decision making [9]. Since 2014, the Nigerian National Malaria Elimination Programme (NMEP) of the Federal Ministry of Health in collaboration with United States President’s Malaria Initiative- Africa Indoor Residual Spray (PMI-AIRS), PMI-VectorLink Project Nigeria, United States Department of Defence/Walter Reed Project, Global Fund, Nigerian Institute of Medical Research (NIMR), Lagos state government and other partners have been involved in the collection of malaria entomology data from sentinel sites established in different states of Nigeria [9, 10]. The initial selection of pioneer malaria vector sentinel sites in six states were done based on a geographic transection of all five ecological zones in Nigeria.

**Fig. 1:**
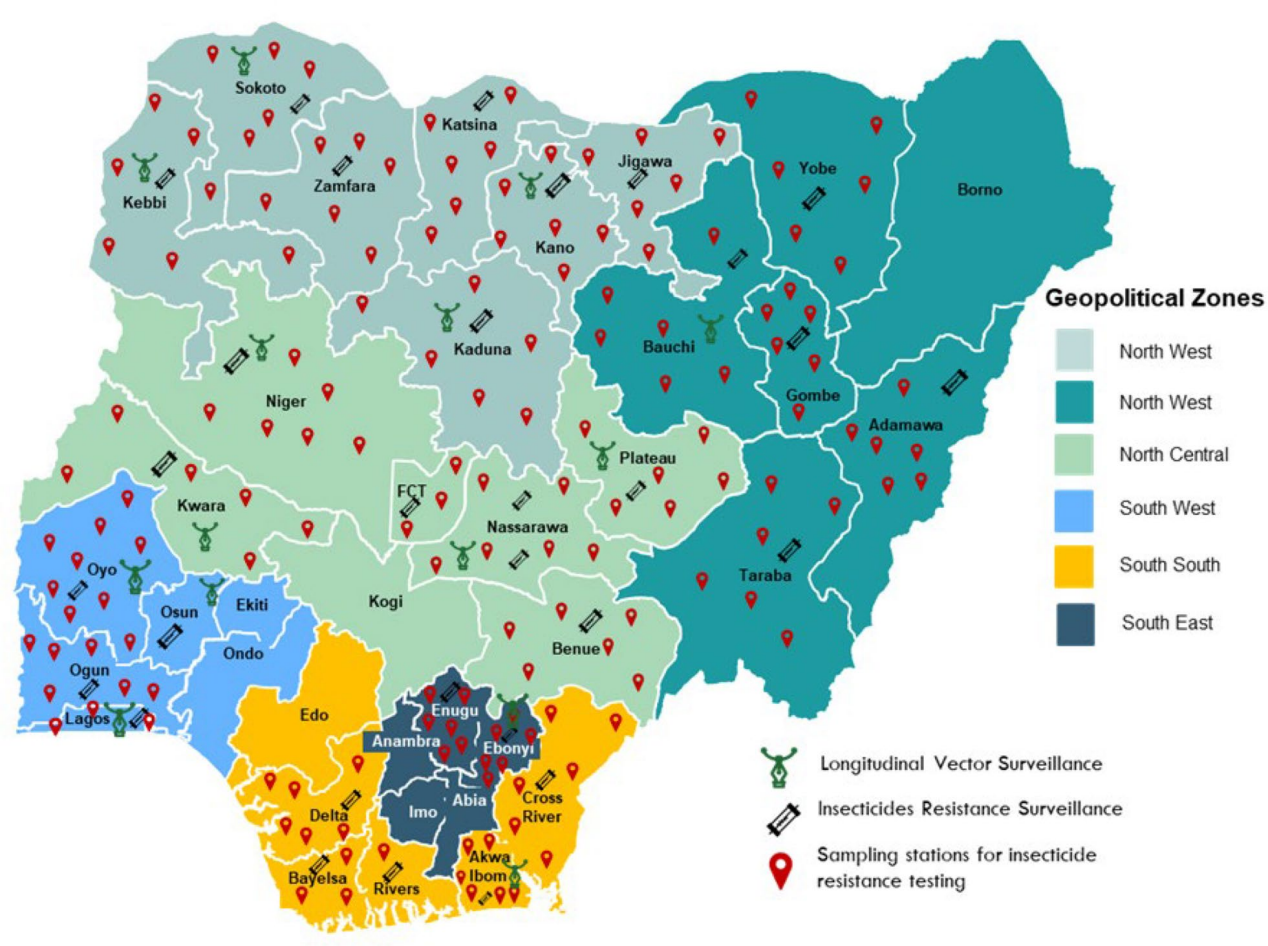
Map of Nigeria showing the malaria vector surveillance and insecticide resistance monitoring sites across states

**Fig. 2:**
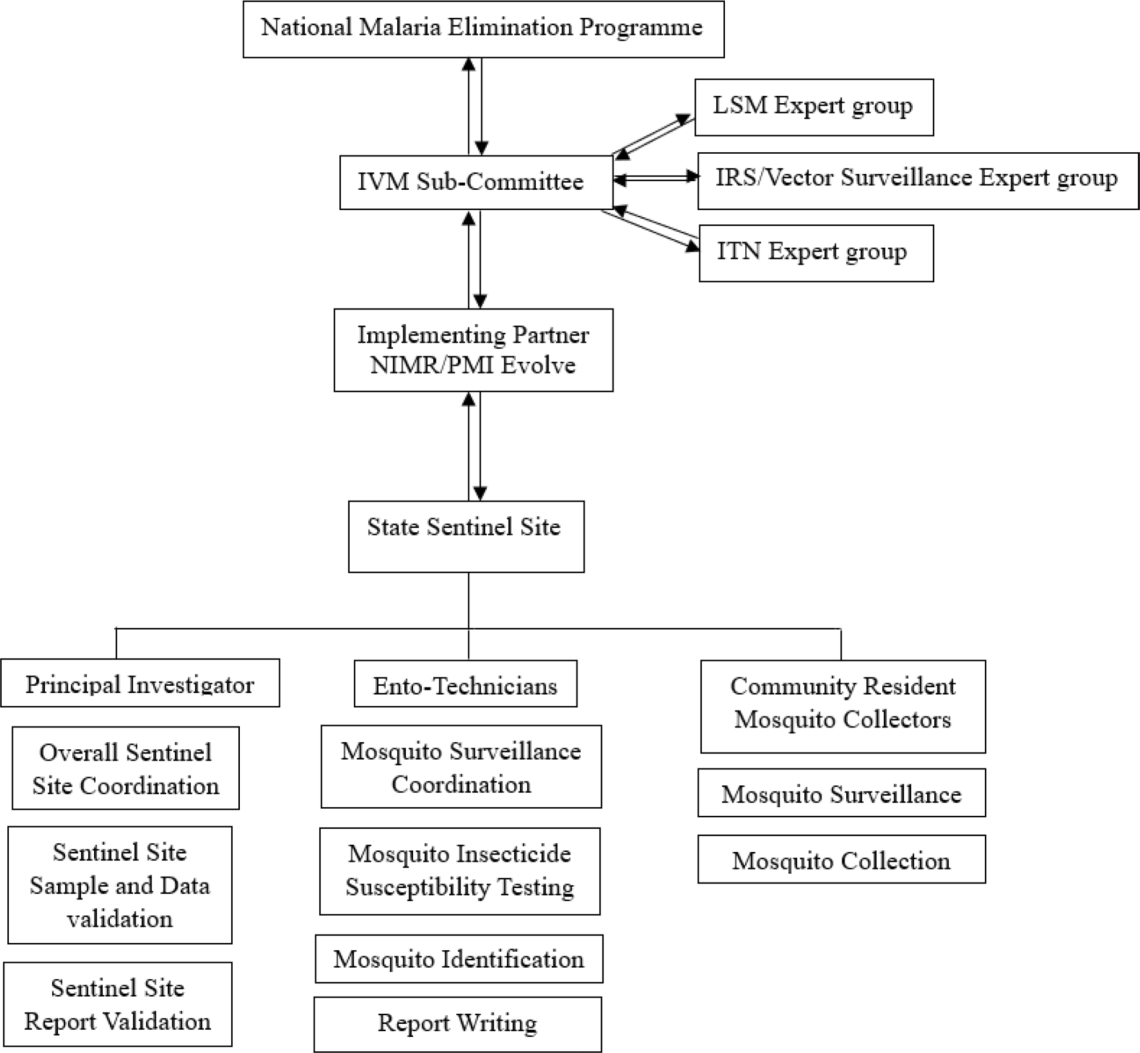
Implementation framework for malaria vector surveillance and insecticide resistance monitoring in Nigeria. *IVM* Integrated Vector Management, *LSM* Larval Source Management, *IRS* Indoor Residual Spraying, *ITN* Insecticide Treated Nets, *NIMR* Nigerian Institute of Medical Research, *PMI* United States President’s Malaria Initiative

Currently, the goal is to establish sentinel sites in all 36 states of the country and Federal Capital Territory for state-specific data-driven vector control [11]. Considerable progress has been made in this regard over the years. Annual insecticide resistance monitoring (IRM) and/or monthly routine malaria vector surveillance (MVS) activities are now ongoing in 29 state-based sentinel sites in the Country (Fig. 1). All the 29 sites carry out IRM while only 14 sites conduct both monthly routine malaria mosquito vector surveillance and insecticide resistance monitoring (Fig. 1). Each sentinel site is officially established by the NMEP, coordinated by the implementing partners and operated by recruited resident personnel from universities/research institutions within the state. The National MVS and IRM implementation framework is summarized in Fig. 2.

## Sentinel site personnel recruitment and training

Trained and experienced malaria entomology staff are required for collection of malaria vector surveillance and IRM data. However, the Nigerian national and state malaria control programmes have limited public health entomology personnel to perform other functions beyond vector control implementation. Personnel capacity and expertise for other functions (vector surveillance, insecticide resistance monitoring and control evaluation) required to support vector control implementation are mostly available within the Nigerian university and research institutes located in different states of the country [7]. Consequently, qualified personnel within universities or research institutions located in the state of sentinel site are selected to serve as principal Investigator (PI) and/or Co-PI leading a team of qualified entomology technicians, community resident mosquito collectors and State Ministry of Health (SMoH) officials to drive the activities of the state sentinel site.

Adverts for the position of PIs are publicized at Federal and State Universities located in the State of sentinel sites and through available entomology and malaria related social media platforms like Entomological Society of Nigeria, Society for Mosquito Control in Nigeria and Public Health and Parasitology Association. Prospective PI candidates are expected to hold a PhD in biological sciences preferably in zoology, entomology or parasitology with evidence of relevant publications and previously demonstrated ability to lead research activities as a PI. Candidates with evidenced proficiency in malaria entomology as supported by institutionalized availability of facilities and basic equipment for entomology work are prioritized over other applicants. The most suitable candidate PI pre-selected, by the NMEP Integrated Vector Management Sub-committee (IVM SC) through the Vector Surveillance/IRS Expert group, is interviewed, nominated and engaged with official letter from NMEP.

Officials of NMEP carry out advocacy visits to the State Government, heads of host universities/research institutions and SMoH to establish institutionalized collaboration among the PI, the SMoH and the host institution for sustainability, safety of equipment and capacity building. Formal institutional agreements are signed to ensure sustainable linkages between the NMEP and collaborating institutions. Practical training and retraining of all personnel engaged are carried out by the NMEP in collaboration with the Nigerian Institute of Medical Research (NIMR), PMI Evolve Project Nigeria and other relevant stakeholders. A list of such trainings conducted for different state sentinel sites is detailed in Table 1.

**Table 1:** Capacity strengthening training workshops conducted on vector surveillance and IRM in Nigeria since 2018

Training programme	Participants	Venue	Date
NMEP-NIMR-GF ento-surveillance training workshop	PIs and ETs from Kano, Niger and Osun states	NIMR Lagos	October, 2018
NMEP-PMI VectorLink ento-surveillance training workshop	PIs and ETs from Cross-river, Kebbi, Zamfara and Sokoto states	PMI Supported Laboratory, Nasarawa State University, Keffi	April, 2019
NMEP-NIMR-GF ento-surveillance training workshop	PIs and ETs from Kwara and Adamawa states	NIMR Lagos	May, 2019
NMEP-PMI VectorLink refresher Entomology training workshop	PIs and ETs from all PMI supported states	Keffi	January, 2020
NMEP-NIMR-GF Ento-surveillance project training workshop	PIs and ETs from Kwara, Osun, Ogun, Delta, Taraba, Gombe, Jigawa, Katsina, Kaduna and Yobe states	NIMR Lagos	July, 2020
NMEP-SuNMaP 2 Ento-surveillance project training workshop	PIs and ETs from Kaduna state	Kaduna state	December, 2020
NMEP-PMI VectorLink Entomology training	PIs and ETs from Bayelsa, FCT and Enugu	Keffi	2021
NMEP-PMI VectorLink Entomology training	PIs and ETs from Sokoto and Kebbi states	Sokoto state	July, 2021
PMI VectorLink Entomology data entry and reporting training workshop	ETs from all PMI states	Keffi	March, 2022
Regional Trainings (Enugu, Keffi and Lagos) to support sampling and identification of *An. stephensi* in Nigeria	Entomology Technicians from PMI and GF Supported sites (29 states)	NSUK, Keffi NAVRC, Enugu, and NIMR, Lagos	Nov. 2022 April 2023
NMEP-NIMR-GF National Ento-surveillance refresher training workshop	PIs and ETs from all GF supported states	NIMR, Lagos	April 2023

*PI* Principal Investigator, *ET* Entomology Technician, *GF* Global Fund, *NSUK* Nasarawa State University, Keffi, *NARC* National Arbovirus and Vector Research Centre, *NIMR* Nigerian Institute of Medical Research, *FCT* Federal Capital Territory, *PMI* U.S. President’s Malaria Initiative, *NMEP* National Malaria Elimination Programme

## Standardization of sentinel site activities and expected outcomes

Material procurement activities are standardized to ensure uniformity across sentinel sites. The same high standard equipment and supplies, such as Centers for Disease Control and Prevention (CDC) light traps, microscopes, mosquito insecticide susceptibility test kits, aerosols and aspirators, needed for vector surveillance activities in each site are centrally procured by NMEP and PMI-Evolve project Nigeria. On an annual basis the PMI supports the NMEP in international procurement of items that can only be sourced outside the country. Personnel team in each vector surveillance site are trained on the appropriate utilization and care of these equipment to ensure safety and efficiency.

Sentinel sites carrying out routine monthly mosquito vector surveillance conduct both Pyrethrum spray catch (PSC) and CDC light trapping of adult mosquitoes monthly following the same standard protocols. The Local Government Areas (LGAs) with the highest malaria prevalence in the state are selected for routine monthly adult mosquito vector surveillance. Only one community with close proximity to major mosquito breeding site within such LGA is selected for the monthly routine adult mosquito vector surveillance. Over a period of the same dates (three days) in each month, indoor and outdoor host-seeking adult mosquito vector collection are conducted in four different houses spread across the community using human-baited CDC light traps. Pyrethrum spray collections of indoor-resting adult mosquitoes (in 36 houses) are carried out in the mornings following overnight collection of host-seeking mosquitoes. The NMEP, through the support of the PMI has pioneered the use of digital tools to capture real time entomological data from the sentinel sites. Mosquito data recorded in the communities are immediately entered into a digital device that registers the data on a platform where sequential review and approval can be done by the PI and data managers.

The annual IRM is conducted in six representative LGAs within each state using the same WHO/CDC procedures and centrally procured materials within the same rainy season period across all state sentinel sites. Mosquito larvae and pupae collected from all available breeding sites within each LGA are brought together, reared to adulthood and tested against the major insecticides (permethrin, deltamethrin, alphacypermethrin, bendiocarb, pirimiphos-methyl, clothianidin, chlorfenapyr) recommended for malaria mosquito vector control. Mosquito samples collected or tested are identified morphologically by skilled entomology technician and validated by the PI of the sentinel site. All samples from the 29 states in the country are sent to NIMR for a second level morphological confirmation and centralized molecular analysis.

The same list of minimum entomological indices/data outcomes to be determined for vector surveillance in each sentinel sites are clearly defined to ensure uniformity of purpose and clarity of vector surveillance direction for the country. The minimum indices to be determined in each site, methodologies to be adopted and justification for their measurements are presented in Table 2. The vector surveillance indices outlined can be grouped broadly into vector density distribution and seasonality, vector behaviour, biting time and location, and vector susceptibility and mechanism of resistance. Vector density distribution and seasonality is covered by indoor resting density and percentage sibling species composition over months and seasons. Human biting rate, biting cycle, parity rate, sporozoite rate, human blood index and entomological inoculation rate all constitute the outcomes under vector behaviour, biting time and location. Vector susceptibility and mechanism of resistance are measured by determining mosquito resistance status, synergist-insecticide resistance status, pyrethroid resistance intensity and knock down resistance frequency.

**Table 2:** List of minimum indicators for entomological surveillance and IRM in Nigeria

Entomological indicators	Definition (methodology)	Justification
Indoor resting density (IRD)	Number of adult female vectors collected indoors per room per day (estimated from PSC collections over a 3 day period)	To determine relative abundance, indoor resting habit and suitability of local vectors for control measures such as IRS and ITNs
Human biting rate (HBR)	Number of *Anopheles* attempting to feed per person per unit time (estimated from CDC-LT collections)	To determine EIR
Biting cycle per location	Number of *Anopheles* attempting to feed per person per hour indoor/outdoor (from CDC-LT collections)	To determine peak biting times indoor and outdoor
Percentage sibling species composition	Percentage of each female *Anopheles* mosquito sibling species in each sentinel site (estimated from morphological and PCR identifications)	To determine species distribution and prevalence
Parity rate (PR)	Proportion of adult female vectors that laid eggs (estimated through ovary dissection)	An indicator for mosquito longevity
Sporozoite rate (SPR)	Proportion of adult female vectors harboring sporozoites in their salivary glands (determined using ELISA/PCR)	To determine EIR
Human blood index (HBI)	Proportion of blood-fed adult female vectors that fed on humans (determined with ELISA/PCR method)	To determine mosquito host preference
Entomological inoculation rate (EIR)	Number of infectious bites by adult female vectors per person per unit time (calculated as product of HBR&SPR)	To estimate the intensity of malaria transmission within the sentinel site
Resistance status	Classification of adult female vectors as confirmed resistant, possible resistant, or susceptible (WHO/CDC bioassay tests) to permethrin, deltamethrin, alphacypermethrin, bendiocarb, DDT, pirimiphos-methyl, clothianidin and chlorfenapyr	To determine response status of *Anopheles* populations in each site to WHO-PQ recommended insecticides
Synergist-insecticide resistance status	Involves pre-exposure to PBO before exposure to insecticide. Full restoration of susceptibility after pre-exposure to the PBO synergist suggests that the metabolic resistance mechanism (enzyme class) related to the synergist plays a role in the insecticide resistance observed If an increase in mortality is not observed, the metabolic mechanisms related to the synergist are likely not involved in the resistance observed	To ascertain the involvement of metabolic resistance mechanism in the expression of resistance by the *Anopheles* mosquito population in each site
Pyrethroid Resistance intensity	Classification of vector populations as having high, moderate, or low pyrethroids (permethrin, deltamethrin, and alphacypermethrin) resistance after WHO/CDC bioassay tests at × 2, × 5 and × 10 concentrations	To determine operational significance of detected pyrethroid resistance
*kdr* frequency	Percentage of *kdr* allele occurrence in the resistant mosquito populations in each sentinel site (determined using PCR)	To determine the role and extent of target site mechanism involvement in DDT & pyrethroid resistance of *An. gambiae* s.l population in each sentinel sites

## Monitoring and evaluation of sentinel site activities

A robust quality assurance supervision system involving monitoring visits by officials of NMEP, NIMR, PMI Evolve and independent consultants occurs periodically in each sentinel site. The monitoring personnel from any of these organizations is adequately funded to travel for on-site supervision of compliance and adherence to relevant surveillance guidelines at the target sentinel site for at least 3 days. Comprehensive report of activities monitored is submitted to NMEP/PMI-Evolve/NIMR for evaluation and further instructions to the sentinel site personnel team. Challenges faced by the sentinel site team are also reported for prompt actions by NMEP and partners.

## Sentinel sites data outcome processing, utilization and storage

Report written by the PI on the data harvested from each sentinel site are analysed and reviewed by the implementing partners (NIMR, PMI-Evolve). The reviewed reports are passed on to the National IRS/Vector Surveillance Expert group for preliminary review following which the report recommendations are submitted to the integrated vector management sub-committee (IVM SC) of the Nigerian NMEP for another review and ratification. The IVM SC comprises NMEP and other implementing partners as well as members of the academia and research institutions. The national decision-making structure (Fig. 2) is such that specific national expert groups (ITN, LSM or IRS/vector surveillance) operating within the IVM SC membership framework consider issues relating to application of sentinel site data to specific vector control strategies. The different expert groups communicate recommendations to the IVM SC for further consideration and adoption by the NMEP. Prior to the establishment of sentinel sites generating routine data, the country lacked a central entomology database.

With the availability of routine entomology data in 29 states in the country, the need to collate and locate the data in a centralized database arose especially in the development of analytics to understand trends and support decision-making. The NMEP and NIMR, with support from Clinton Health Access Initiative (CHAI) and PMI-Evolve Nigeria, have developed a national entomological database as a module on the National Malaria Data Repository-NMDR. This provides a platform where entomology data are collated and reported for easy access to government, partners, and research institutions.

## Practical applications of sentinel site data outcomes to inform policy

Before the establishment of state sentinel sites, mass distribution of pyrethroid LLINs has been ongoing in Nigeria with periodic replacement campaigns for the protection of all households especially in the rural communities. Currently ITNs remain the major primary vector control tool in the country; between 2015 and 2018, with support from several partners, the Nigerian government has distributed over 127.9 million ITNs in 32 states through mass distribution campaigns and another 16.3 million ITNs through other routine systems and channels [12].

Earlier, local state surveillance and IRM data were not available to inform the specific type of ITN to be procured for each state. Now, state-based ITN procurement is driven by entomological data from the state sentinel sites. For instance, ITN policy change from conventional to Piperonyl Butoxide (PBO) nets have been implemented after consistent records of high level pyrethroid resistance coupled with pyrethroid-PBO synergist susceptibility in Ebonyi and Oyo states. The ITN policy change in Ebonyi represented the first programmatic deployment of PBO ITNs in Nigeria in November 2019. In recent times, the conventional standard ITNs usually procured for some states like Oyo, Kebbi and Cross Rivers have also been replaced by PBO and Interceptor G2 dual active ingredients (AI) nets.

## Analysis of the national MVS programme and future directions

Establishments of sentinel sites in the remaining states of the country is necessary to ensure the use of appropriate data for the control of malaria vectors in each state of the country. Beyond this, global attention is currently shifting to district/community-based vector control [13] requiring targeted district/community-based vector surveillance and insecticide resistance monitoring. This is due to the significant spatial heterogeneities observed in the entomological indices of malaria transmission across limited distances [14]. Thus, the establishment of state-based sentinel sites in Nigeria should only be the beginning of efforts to extend such to the district and community level. Annual insecticide resistance monitoring should be extended from six representative LGAs per state to all the LGAs/districts in each state. The one community per state monthly routine mosquito vector surveillance should also be extended to at least one representative community per district or LGA. This will increase the precision level of targeted malaria vector control carried out against malaria vectors in specific districts or communities within the country.

Generally, the cost of malaria control in Nigeria is enormous. For the period 2020–2023, preliminary analysis by Nigerian NMEP showed that US$ 2.75 billion is needed to achieve high coverage of malaria control interventions in targeted areas, and full availability of diagnosis and treatment in public health facilities [3]. It is therefore critical to ensure that these interventions are evidence-based to avoid the waste of these resources and efforts. Data from annual IRM activity guides decisions on the type of ITN to be procured for each state. This probably explains the prioritization of IRM in all the existing 29 state sentinel sites. The entomological indices listed as the minimum to be determined in each state sentinel site is comprehensive considering the indices outlined in the WHO guidelines for malaria entomology and vector control [15]. However, the implementation of routine monthly mosquito vector surveillance only in 14 out of the 36 states in the country is still a major limitation. This indicates that data on host-seeking vector species composition, distribution, seasonality, biting time and location required for targeted adult malaria vector control in the remaining states are not currently available. These data are required to assess the performance of deployed ITNs and to reveal the possible need for complementary control measures. Apparently, the Nigerian NMEP needs to intensify fund mobilization efforts for expanded vector surveillance and IRM activities in the country. One way is to improve advocacy to state and local governments on the critical need to own and fund expanded vector surveillance within their domain. Already, funding for malaria control in Nigeria comes from Global Fund to Fight HIV AIDS, Tuberculosis and Malaria, U.S. President’s Malaria Initiative (PMI), World Bank (WB), Islamic Development Bank (IDB), World Health Organization (WHO), United National Children’s Fund (UNICEF), Bill & Melinda Gates Foundation (BMGF), Clinton Health Access Initiative (CHAI), GiveWell Community Foundation (GWCF), Against Malaria Foundation (AMF), Nigeria Liquefied Natural Gas Company (NLNG), Alliance for Malaria Prevention (AMP) and others [16].

The multi-layered structure of data report review, validations and decision-making from the sentinel site PI to the implementing partners, IRS/Vector Surveillance Expert group and IVM SC is highly commendable and necessary to ensure limitations of errors. Real time capture of entomological data on digital platforms allows for the immediate flagging of inconsistent data. Field workers viewing this can quickly consider the cause of error, offer explanations or make adjustments while still on the field. This ensures accountability and transparency in field mosquito data collection in the different sentinel sites all across the country.

Practical applications of state-based evidence for ITN procurement represent a major departure from the earlier distribution of conventional pyrethroid nets in all states regardless of the differences in mosquito pyrethroid insecticide susceptibility status. States in Nigeria with the highest malaria prevalence; Kano 26%, Katsina 29%, Bauchi 32%, Sokoto 36% and Kebbi 49% [17] have highest numbers of LGAs (Kano 44; Katsina 34; Bauchi 20, Sokoto 23; Kebbi 21). District/LGA based ITN procurement guided by the LGA IRM data should now be encouraged to prevent the costly assumptions associated with the use of data from only 6 LGAs for all LGAs in each state.

Personnel capacity for routine mosquito vector surveillance is limited in most countries at risk of vector-borne diseases [8]. However, the current engagement and regular training of vector personnel in different state sentinel sites in Nigeria is a major step towards malaria entomology capacity building in all the zones within the country. Moreover, the community residents engaged as mosquito collectors could drive the implementation of district/community-based vector surveillance activities when executed across the country.

## Conclusions

The described harmonized Nigerian MVS system engaging sentinel site PIs, Ento-technicians, SMoH officials and community-based mosquito collectors across different states of the country is expected to generate a critical mass of malaria vector research personnel over time. The institutionalization of malaria vector surveillance across research and policy organizations will further help to galvanize vector control support towards significant malaria burden decline in malaria worse-hit Nigeria. Other countries with high malaria transmission risks could consider the engagements of resident university/institute researchers as well as the regular community dwellers as part of collective national capacity building and stakeholder mobilization towards malaria elimination. Focus of these countries should be on generating and utilizing community/district based entomological data for community/district based malaria vector control. Malaria vector control authorities in malaria endemic countries, especially in Africa, should keep the focality of malaria transmission in mind and prioritize sustainable partnerships with community residents.

## Data Availability

Not applicable.

## Abbreviations

USAID: United States agency for international development
PMI: United States president’s malaria initiative
WHO: World Health Organization
LLIN: Long-lasting insecticidal nets
IRS: Indoor residual spraying
MVS: Malaria vector surveillance
IRM: Insecticide resistance monitoring
NMEP: National malaria elimination programme
GF: Global fund to fight AIDS, tuberculosis and malaria
FCDO: Foreign, commonwealth and development office
DFID: Department for international development
AIRS: Africa indoor residual spray
FCT: Federal capital territory
PI: Principal investigator
Co-PI: Co-principal investigator
SMoH: State ministry of health
NIMR: Nigerian institute of medical research
ET: Entomology technicians
CDC: Centers for disease control and prevention
IRD: Indoor resting density
PSC: Pyrethrum spray catch
HBR: Human biting rate
CDC LTs: CDC human-baited light traps
PR: Parity rate
SPR: Sporozoite rate
ELISA: Enzyme-linked immunosorbent assay
PCR: Polymerase chain reaction
HBI: Human blood index
EIR: Entomological inoculation rate
DDT: Dichlorodiphenyltrichloroethane
PBO: Piperonyl butoxide
Kdr: Knock down resistance
IVM SC: Integrated vector management subcommittees
LSM: Larval source management
FMoH: Federal ministry of health
TWG: Technical working group
ITN: Insecticide treated nets
LGA: Local government area
NAMRU: Naval medical research Unit
CHAI: Clinton health access initiative
NMDR: National malaria data repository
AMP: Alliance for malaria prevention
NMIS: Nigeria malaria indicator survey
